# Carbonyl Reduction of Flubendazole in the Human Liver: Strict Stereospecificity, Sex Difference, Low Risk of Drug Interactions

**DOI:** 10.3389/fphar.2019.00600

**Published:** 2019-05-28

**Authors:** Vladimír Kubíček, Lenka Skálová, Adam Skarka, Věra Králová, Jana Holubová, Jana Štěpánková, Zdeněk Šubrt, Barbora Szotáková

**Affiliations:** ^1^Department of Biophysics and Physical Chemistry, Faculty of Pharmacy in Hradec Králové, Charles University, Hradec Králové, Czechia; ^2^Department of Biochemical Sciences, Faculty of Pharmacy in Hradec Králové, Charles University, Hradec Králové, Czechia; ^3^Department of Chemistry, Faculty of Science, University of Hradec Králové, Hradec Králové, Czechia; ^4^Department of Biology, Faculty of Medicine in Hradec Králové, Charles University, Hradec Králové, Czechia; ^5^Department of Surgery, Faculty of Medicine in Hradec Králové, Charles University, Hradec Králové, Czechia; ^6^Department of Surgery, University Hospital Hradec Králové, Hradec Králové, Czechia

**Keywords:** flubendazole, carbonyl reduction, human, enzyme kinetics, stereospecificity, sex difference

## Abstract

Flubendazole (FLU), an anthelmintic drug of benzimidazole type, is now considered a promising anti-cancer agent due to its tubulin binding ability and low system toxicity. The present study was aimed at determining more information about FLU reduction in human liver, because this information has been insufficient until now. Subcellular fractions from the liver of 12 human patients (6 male and 6 female patients) were used to study the stereospecificity, cellular localization, coenzyme preference, enzyme kinetics, and possible inter-individual or sex differences in FLU reduction. In addition, the risk of FLU interaction with other drugs was evaluated. Our study showed that FLU is predominantly reduced in cytosol, and the reduced nicotinamide adenine dinucleotide phosphate (NADPH) coenzyme is preferred. The strict stereospecificity of FLU carbonyl reduction was proven, and carbonyl reductase 1 was identified as the main enzyme of FLU reduction in the human liver. A higher reduction of FLU and a higher level of carbonyl reductase 1 protein were found in male patients than in female patients, but overall inter-individual variability was relatively low. Hepatic intrinsic clearance of FLU is very low, and FLU had no effect on doxorubicin carbonyl reduction in the liver and in cancer cells. All these results fill the gaps in the knowledge of FLU metabolism in human.

## Introduction

Anthelmintic drugs of the benzimidazole type are often used in human and veterinary medicine. They have broad anthelmintic activity, low toxicity, and, therefore, a small incidence of undesirable effects. The mechanism of their action is based on specific binding to the microtubule subunit tubulin, resulting in the disruption of the microtubule structure and the inhibition of secretory vesicle transport. In addition, these anthelmintics also inhibit glucose uptake, exhaust glycogen stores, and prevent ATP formation, leading to the death of the helminth. The ability to prevent microtubule formation and glucose uptake predisposes the benzimidazole anthelmintics toward a possible cytostatic action, because tubulin is essential for cell division, and tubulin is a target structure of many anticancer drugs, including paclitaxel, colchicine, and vinca alkaloids (Hanusova et al., 2015; Yadav et al., 2016).

Flubendazole, FLU, [5-(4-fluorobenzoyl)-1*H*-benzimidazol-2-yl]-carbamic acid methyl ester, is a benzimidazole-type anthelmintic drug approved for helminthosis treatment in humans, pigs, dogs, and poultry. FLU exhibits low toxicity in all target species; however, this fact could be based at least partly on low bioavailability of FLU in mammals, as orally bioavailable amorphous solid dispersion formulation of FLU showed some toxicity at high doses (Lachau-Durand et al., 2019). Several studies have shown an anti-proliferative effect of FLU in many *in vitro* and *in vivo* models. Compared with other anticancer drugs, which have a similar mechanism of action, such as vinca alkaloids, the advantage of FLU is the fact that it is not a substrate for P-glycoprotein. Therefore, there is less risk of developing resistance, as well as the absence of serious undesirable effects, such as peripheral neuropathy (Spagnuolo et al., 2010; Kralova et al., 2013; Michaelis et al., 2015; Kralova et al., 2016). In addition to potential use of FLU as an anticancer drug, FLU is also considered as a macrofilaricide (Geary et al., 2019). Because FLU has been registered in Europe for treatment of gut-residing nematodes in humans (Mackenzie and Geary, 2011), its repurposing could be expected soon.

FLU metabolism has been tested in several animal species, for example, rats, pigs, cattle, and sheep. In the liver, FLU is metabolized *via* carbonyl reduction leading to reduced FLU (FLUR) and *via* hydrolysis to decarbamoylated FLU (Virkel et al., 2012; Mate et al., 2017). Both FLU metabolites have shown a significantly lower anthelmintic activity than the parent drug (Bartikova et al., 2010). In *in vitro* metabolic studies with rat liver microsomes, a decarbamoylated metabolic product from FLUR has been also formed (Mate et al., 2008; Ceballos et al., 2015). In human cancer cell lines, FLU undergoes carbonyl reduction and in some cases also subsequent FLUR methylation (Stuchlikova et al., 2018). Surprisingly, in our previous study, when FLU was incubated with precision-cut human liver slices, only one FLU metabolite, FLUR, was detected (Stuchlikova et al., 2018). In human liver microsomes incubated with FLU, FLUR as well as decarbamoylated FLU, was found (Mate et al., 2017). Nevertheless, in precision-cut liver slices from all four human donors, neither hydrolysis nor methylation of FLU or FLUR was detected using sensitive UHPLC/MS analysis (Stuchlikova et al., 2018). Based on these facts, carbonyl reduction is the major or possibly the only metabolic pathway of FLU in the human liver. No information has been reported regarding FLU reduction (e.g., efficiency, localization, responsible enzymes, sex differences, inter-individual differences). Because FLU has been used in human medicine for several years, this information is vitally important for continuing safe treatment with FLU.

For this reason, the present study focused on finding more information about FLU reduction in human hepatic subcellular fractions. Stereospecificity, cellular localization, coenzyme preference, and enzyme kinetics were studied. Liver samples from 12 patients (6 male and 6 female patients) were used to follow up on possible inter-individual and sex differences in FLU reduction. In addition, the effect of FLU on the carbonyl reduction of the common cytostatic drug doxorubicin (DOX) was also studied with the aim of evaluating possible drug–drug interactions.

## Materials and Methods

FLU was purchased from Janssen Pharmaceutica (Prague, Czechia). Recombinant human carbonyl reductase 1 (CBR1) was obtained from MyBioSource (San Diego, CA, USA). Albendazole, menadione (MEN), luteolin (LUT), DOX, reduced nicotinamide adenine dinucleotide phosphate (NADPH), and NADPH, were purchased from Sigma-Aldrich (Prague, Czechia). All other chemicals used were of HPLC or analytical grade.

### Human Liver Samples

This study was carried out in accordance with the recommendations of the Declaration of Helsinki and Good Clinical Practice Guidelines. The protocol was approved by the Ethics Committee of University Hospital Hradec Králové, Czechia (project “Collection of liver tissue for isolation of hepatocytes, preparation of precision liver slices and subcellular fractions”, No. 201703 S14P). All patients gave written informed consent in accordance with the Declaration of Helsinki. Small pieces of healthy liver tissue were obtained from 12 patients (6 male patients, 35–81 years old, and 6 female patients, 45–73 years old) undergoing liver surgery due to a tumor. Liver samples were instantly put into a chilled vessel with Euro‐Collins solution and transported to the laboratory, where the liver tissue was frozen in dry ice and stored in a freezer (−80°C) until processing.

### Cultivation of Cancer Cells SW480

Human colon adenocarcinoma cell line SW480 was purchased from ATCC (CCL-228; passage 146; LGC Standards, Poland). The cells were multiplied in three passages, frozen in aliquots, and stored in liquid nitrogen. The absence of mycoplasma was periodically checked by Generi Biotech (Hradec Králové, Czech Republic). The SW480 cells were maintained in DMEM medium supplemented with 10% heat-inactivated fetal bovine serum and 1% penicillin/streptomycin. The cells were grown in a humidified atmosphere of 5% CO_2_ at 37°C. When confluence was reached, the cells were scraped into cooled 0.1 M sodium phosphate buffer, pH 7.4 and used for subcellular fractions preparation.

### Preparation of Subcellular Fractions

Frozen pieces of liver were thawed at room temperature for up to 15 min and individual livers were homogenized in a 0.1 M sodium phosphate buffer, pH 7.4 at the ratio of 1:6 (v/v), using a Potter-Elvehjem homogenizer and sonication with Sonopolus (Bandelin, Germany). The suspension of the SW480 cells was homogenized using sonication only. The subcellular fractions were isolated by differential centrifugation of the homogenates (Gillette, 1971) and stored at −80°C. Protein concentrations were assayed using the bicinchoninic acid (BCA) assay according to the manufacturer’s instructions (Sigma-Aldrich).

### Biotransformation of Flubendazole *In Vitro*

The cytosolic or microsomal fraction was incubated with FLU (10 μM). The reaction mixture (total volume of 0.3 ml) contained 100 μl of subcellular fraction containing 0.4 to 0.6 mg of proteins, substrate pre-dissolved in dimethyl sulfoxide (1% concentration in reaction mixture), 1 mM NADPH or 1 mM NADH and 0.1 M Na-phosphate buffer, pH 7.4. Two types of blank samples were prepared, without substrate (biological blank) and without the subcellular fraction (chemical blank). In a kinetic study, a concentration range of 0 to 10.0 μM of FLU, 1 mM NADPH or 1 mM NADH and 100 μl of cytosol were used. Inhibition of the FLU reduction was carried out by incubation of the cytosol with FLU (10 μM) in the presence of specific inhibitors (LUT, MEN) of CBR1 (range, 0–50 µM). All incubations were carried out at 37°C for 30 min under aerobic conditions. The product formation was linear up to 60 min. At the end of incubation, 30 μl of ammonium solution (concentrated), 30 μl of internal standard (10 µM albendazole), and 700 μl of ethyl acetate were added, shaken (3 min, vortex), and centrifuged (5 min, 10,000×*g*). Supernatants were evaporated and stored under −20°C until HPLC analyses.

### Incubation of Doxorubicin *In Vitro*

The cytosol from the human livers and cancer cells SW480 was incubated with 20 µM DOX with or without FLU (2 and 10 μM) in the presence of 1 mM NADPH in 30 mM Tris–HCl buffer, pH 7.4. The reaction mixtures (100 μl) were incubated for 30 min at 37°C. The reactions were stopped with the addition of 100 μl of ice-cold acetonitrile and 100 μl of ice-cold methanol; the samples were shaken (3 min, vortex) and centrifuged (5 min, 10,000×*g*). Supernatants were stored under −80°C until HPLC analyses.

### HPLC Analysis

**Figure 1:**
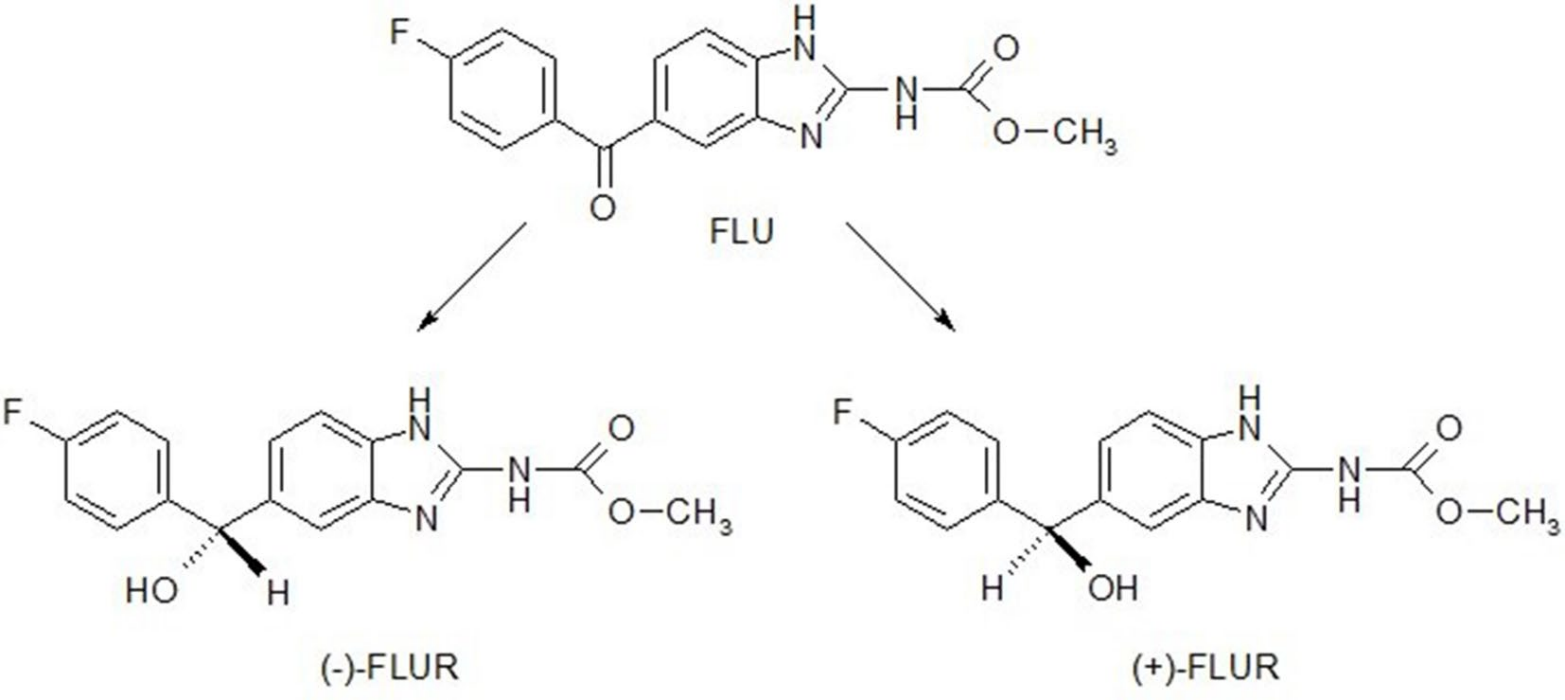
Structural formulae of flubendazole (FLU) and enantiomers of its reduced metabolite FLUR, (+)-FLUR enantiomer and (−)-FLUR enantiomer.

**Figure 2:**
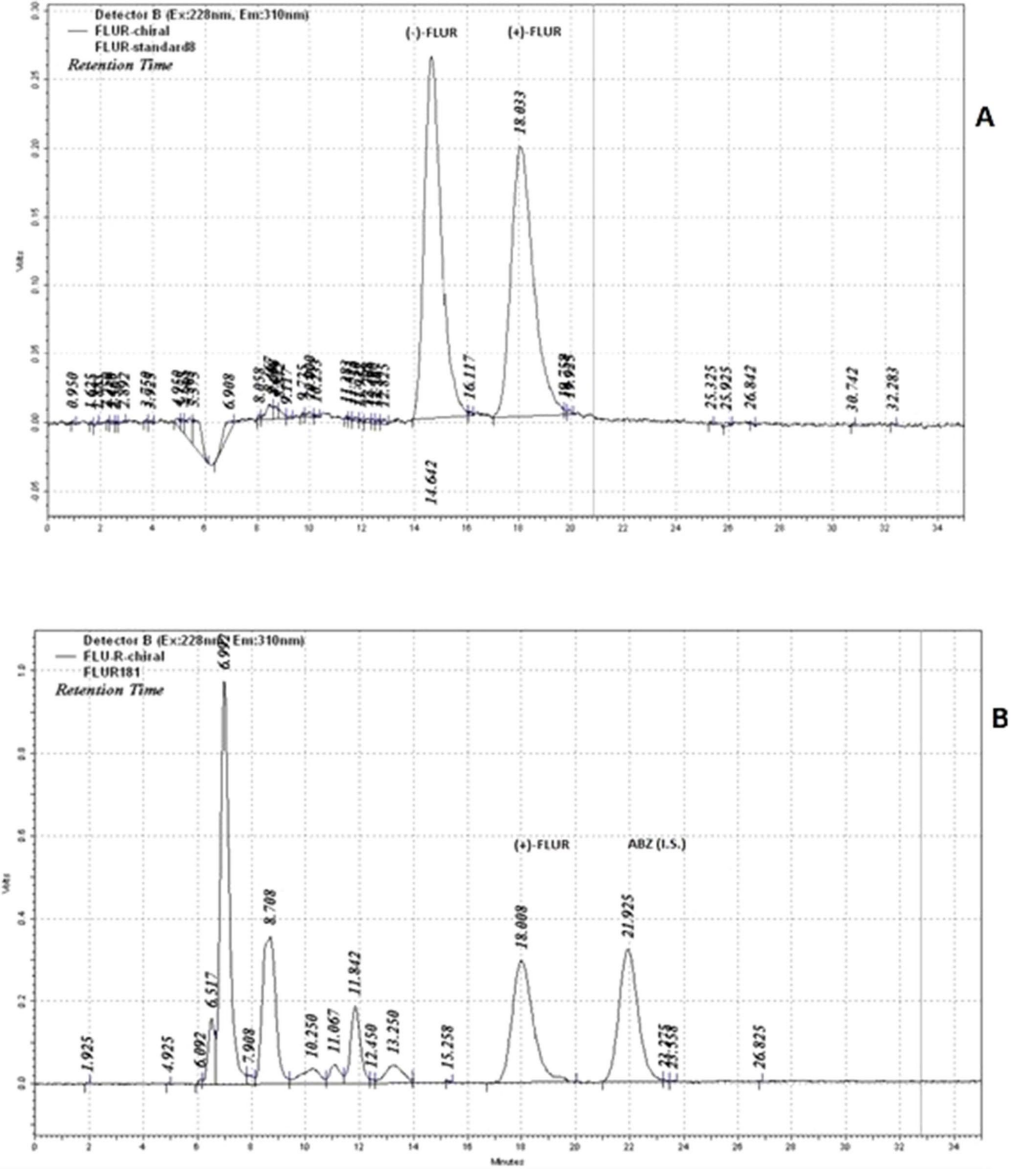
Chiral chromatograms of standard of FLUR enantiomers **(A)**, and incubation sample **(B)** (pooled human liver cytosol incubated with 10 µM FLU). Retention times of the individual compounds: *t*_R_ = 14.6 min for (−)-FLUR enantiomer; *t*_R_ = 18.0 min for (+)-FLUR enantiomer; *t*_R_ = 21.9 min for I.S. albendazole (ABZ).

Details concerning the HPLC analyses including validations were described in Nobilis et al. (2008). A chiral HPLC analysis was employed for the determination of reduced FLU (FLUR) enantiomers—(+)-FLUR and (−)-FLUR; concentrations of FLU, (+)-FLUR, and (−)-FLUR in the samples were determined using an internal standard method (I.S., albendazole). A 250 × 4.6 mm chromatographic column packed with Chiralcel OD-R (Daicel, Japan) and a mobile phase containing methanol, 1 M aqueous NaClO_4_ (75: 25, v/v, pH 6.85) at a flow rate of 0.5 ml·min^−1^ in isocratic mode enabled a satisfactory separation of all analytes within 25 min. Retention times of FLUR enantiomers were *t*_R_ = 14.6 min for (−)-FLUR enantiomer and *t*_R_ = 18.0 min for (+)-FLUR enantiomer. A tandem of an UV photodiode-array and a fluorescence detector was used for the determination of the abovementioned benzimidazole compounds. The structures of FLU and FLUR enantiomers are shown in **Figure 1**. The typical chiral chromatograms of FLUR standards and sample are presented in **Figure 2**.

DOX was detected on the UHPLC Agilent 1290 Series chromatographic system equipped with Zorbax C18 Eclipse Plus (2.1 × 50 mm, 1.8 μm) column with 1290 Infinity inline filter (Agilent). The HPLC method used by Skarka et al. (2011) was adapted to the UHPLC system: isocratic elution of 1.0 ml·min^−1^ by 0.1% formic acid in water and acetonitrile in the ratio 76:24; 30°C thermostated column compartment; fluorescence detection at λ_ex_ = 480 nm and λ_em_ = 560 nm.

### Enzyme Assay

The human recombinant CBR1 activity was assayed using a slight modification of previously published method (Mate et al., 2008). The assay was performed in four replicates and was repeated three times. The amount of organic solvent in the final reaction mixture did not exceed 1% (v/v). The activity of CBR1 was assessed using 10-μM concentration of the substrates, MEN, DOX, and FLU. The consumption of NADPH in the reaction mixture, which served for the assessment of the reductase activity, was determined spectrophotometrically at 340 nm at 37°C using the microplate reader Tecan Infinite M200.

### Western Blot Analysis

Cytosolic proteins of the individual human livers (30 μg in each lane) were separated by sodium dodecyl sulfate–polyacrylamide gel electrophoresis (SDS-PAGE) and subsequently transferred onto nitrocellulose membranes (0.45 μm) using a Trans-Blot^® ^Turbo™ Transfer System (Bio-Rad, USA). The membranes were blocked in 5% non-fat dry milk/TBS-Tween-20 for 2 h. For the immunodetection of CBR1, the membranes were probed overnight with primary goat polyclonal antibody to CBR1 (1:1000, ab4148; Abcam, Cambridge, UK) diluted in TBS-Tween 20 supplemented with 1% BSA, washed four times with a TBS-Tween 20 buffer, and probed with a secondary antibody (bovine anti-goat IgG-HRP, 1:3000, Santa Cruz Biotechnology, USA) for 1 h. The signal was detected using an enhanced Amersham ECL chemiluminescence kit (GE Healthcare Life Sciences, USA) according to the manufacturer’s instructions. β-Actin (mouse monoclonal, 1:3,000, Abcam) served as the loading control. The intensity of bands was evaluated using a C-DiGit™ Blot Scanner (LICOR Biotechnology, USA). Protein levels were measured in two independent experiments.

### Statistical Analysis

All experiments were repeated three times, with at least four technical replicates performed each time. The calculations were done using Microsoft Excel and GraphPad Prism 7.04. Statistical significance was tested by a one-way ANOVA and Welch’s t test, and differences were considered statistically significant at p < 0.05. Data are presented as the mean ± standard deviation.

## Results

### Preference of Coenzyme and Stereospecificity of Flubendazole Reduction

The *in vitro* biotransformation studies of FLU with pooled human liver cytosolic and microsomal fractions were designed to determine the preference of coenzymes for FLU reduction and stereospecificity of this process. The identification and determination of FLU and enantiomers of reduced FLU, (+)-FLUR and (−)-FLUR, in the incubation mixtures was undertaken using the chiral HPLC method.

FLU reduction was highest in the cytosol with the NADPH coenzyme; on the other hand, in microsomal fraction with NADH coenzyme, no FLUR was detected (**Table 1**). The reduction of the carbonyl group of FLU was shown to be strictly stereospecific in the human liver, as only the (+)-FLUR enantiomer was found.

**Table 1:** Reduction of flubendazole in the pooled human liver subcellular fractions of both sexes.

	(+)-FLUR [pmol/mg protein]	(−)-FLUR [pmol/mg protein]
CYT NADPH	31.8 ± 0.6	0
CYT NADH	14.7 ± 0.9	0
MIC NADPH	1.3 ± 0.5	0
MIC NADH	0	0

The values represent the mean ± standard deviation of three independent experiments.

FLUR, reduced flubendazole; (+)-FLUR, (+)-enantiomer of FLUR; (−)-FLUR, (−)-enantiomer of FLUR; CYT, pooled cytosol; MIC, pooled microsomes.

### Kinetics of Flubendazole Reduction *In Vitro*

**Figure 3:**
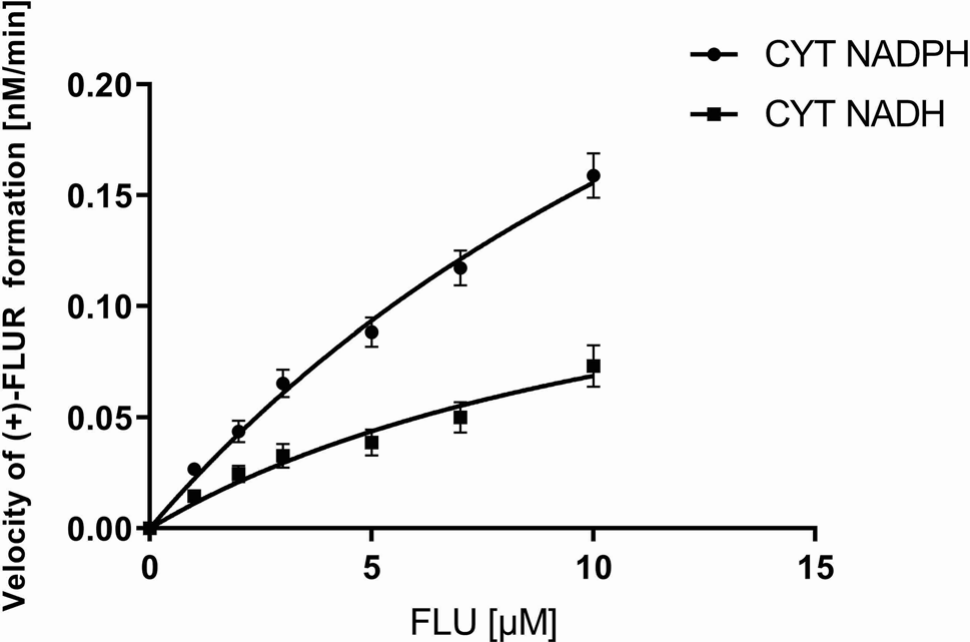
Kinetics of flubendazole (FLU) reduction in pooled human liver cytosolic fraction of both sexes (CYT). CYT was incubated with 1.0 to 10.0 µM FLU with coenzyme NADPH or NADH. The values represent the mean ± standard deviation of three independent experiments.

The pooled human liver cytosol was incubated with various concentrations of FLU (0–10 μM). The amount of (+)-FLUR formed in the incubation mixture was expressed as a reaction velocity. The direct plot of the reaction velocity versus substrate concentration fit the Michaelis–Menten equation (**Figure 3**) well. Using GraphPad Prism 7.04 software, the values of the kinetic parameters, apparent maximal velocity V′_max_ and apparent Michaelis constant K′_m_, were calculated. The apparent maximal velocity V′_max_ indicates the rate of product formation at enzyme saturation with the substrate. The apparent Michaelis constant K′_m_ expresses the affinity of enzymes toward the substrate in the multi-enzymatic system. The ratio of these kinetic parameters (V′_max_/K′_m_) represents enzymatic efficiency, the so-called intrinsic clearance (Cl_int_). The apparent maximal velocity V′_max_ and apparent Michaelis constant K′_m_ for NADPH coenzyme were higher than those for the NADH coenzyme. Hepatic intrinsic clearance of FLU is very low (**Table 2**).

**Table 2:** Kinetic parameters obtained for (+)-FLUR formation in the pooled human liver cytosol of both sexes with NADPH or NADH coenzymes.

	CYT NADPH	CYT NADH
V’_max_ [nM·min^−1^]	0.466 ± 0.043	0.161 ± 0.024
K’_m_ [µM]	19.91 ± 2.49	13.34 ± 3.07
Cl_int_ [min^−1^]	0.000023	0.000012

The values represent the mean ± standard deviation of three independent experiments.

(+)-FLUR, (+)-enantiomer of reduced flubendazole; V′_max_, apparent maximal velocity of the reaction; K′_m_, apparent Michaelis constant; Cl_int_, intrinsic clearance; CYT, pooled human liver cytosol of both sexes.

### Inter-Individual Differences in Flubendazole Reduction in Human Hepatic Cytosol

**Figure 4:**
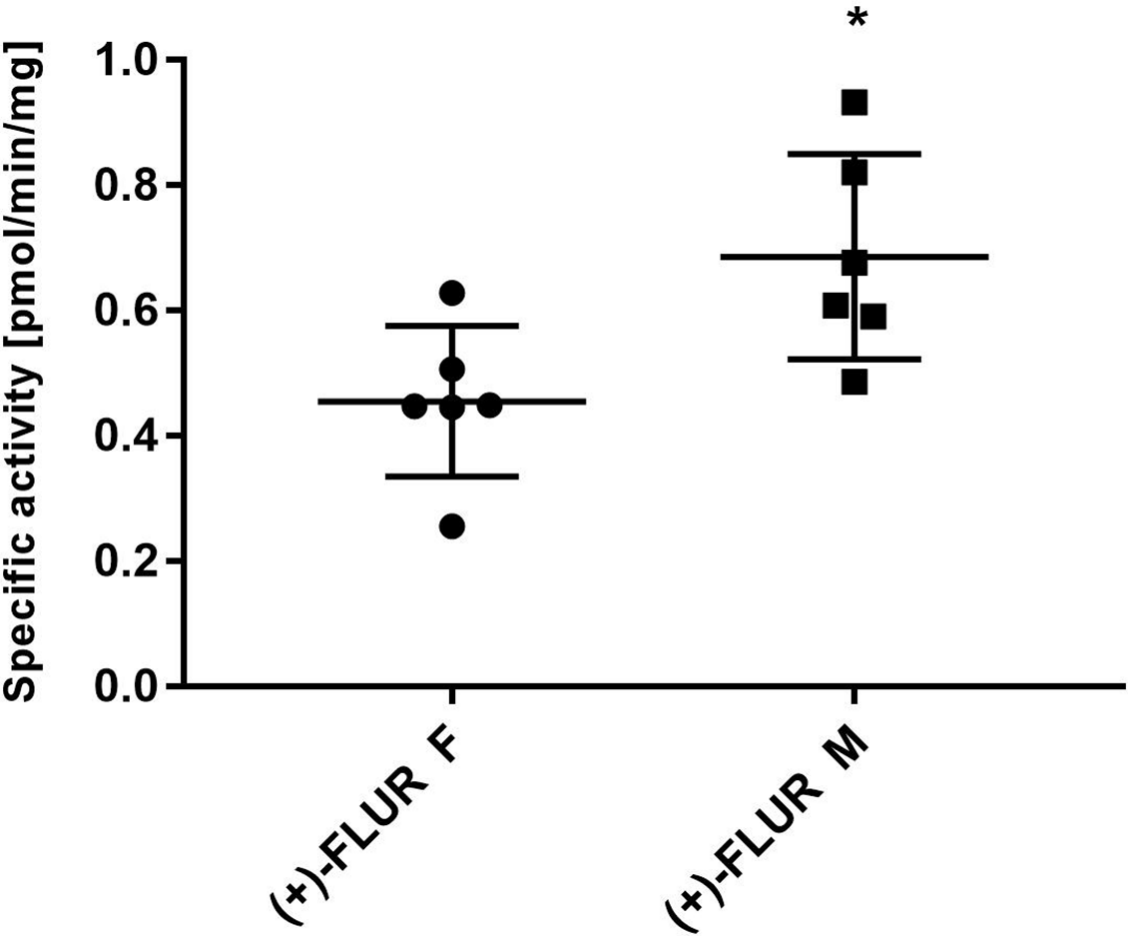
Inter-individual differences in flubendazole (FLU) reduction and gender differences. Liver cytosols of individual male patients (n = 6) and female patients (n = 6) were incubated with 10 µM FLU. The values represent the mean ± standard deviation of three independent experiments. (+)-FLUR enantiomer of reduced FLU; F, female; M, male, *Significantly different compared with F.

The individual human liver cytosols (six male and six female patients) were incubated with 10 μM FLU. After incubation, the amount of (+)-FLUR formed in the incubation mixture was analyzed and specific activity was calculated. Significantly higher specific activity of reductases toward FLU was found in male patients compared to female patients (**Figure 4**), but no significant inter-individual differences in the group of males and females were found.

### Effect of CBR1 Inhibitors on Flubendazole Reduction

Two inhibitors of carbonyl reductase 1 (CBR1) were used to determine the involvement of CBR1 in FLU reduction. The pooled human liver cytosol was incubated with 10 µM FLU and NADPH coenzyme in the presence of different concentrations of MEN or LUT. Both MEN and LUT inhibited FLU reduction in cytosol, with MEN a stronger inhibitor than LUT. The 50% inhibition concentration (IC_50_) values were calculated for both inhibitors. The obtained IC_50_ values and their 95% confidence intervals are given in **Table 3**.

**Table 3:** Inhibition concentration (IC_50_) of luteolin and menadione to flubendazole reduction in the pooled human liver cytosol of both sexes.

	LUT	MEN
IC_50_ (µM)	8.21 ± 3.71	0.34 ± 0.26

The values represent the mean ± standard deviation of three independent experiments.

LUT, luteolin; MEN, menadione.

### Specific Activity of Human Recombinant CBR1 Toward Flubendazole and Other Substrates

**Figure 5:**
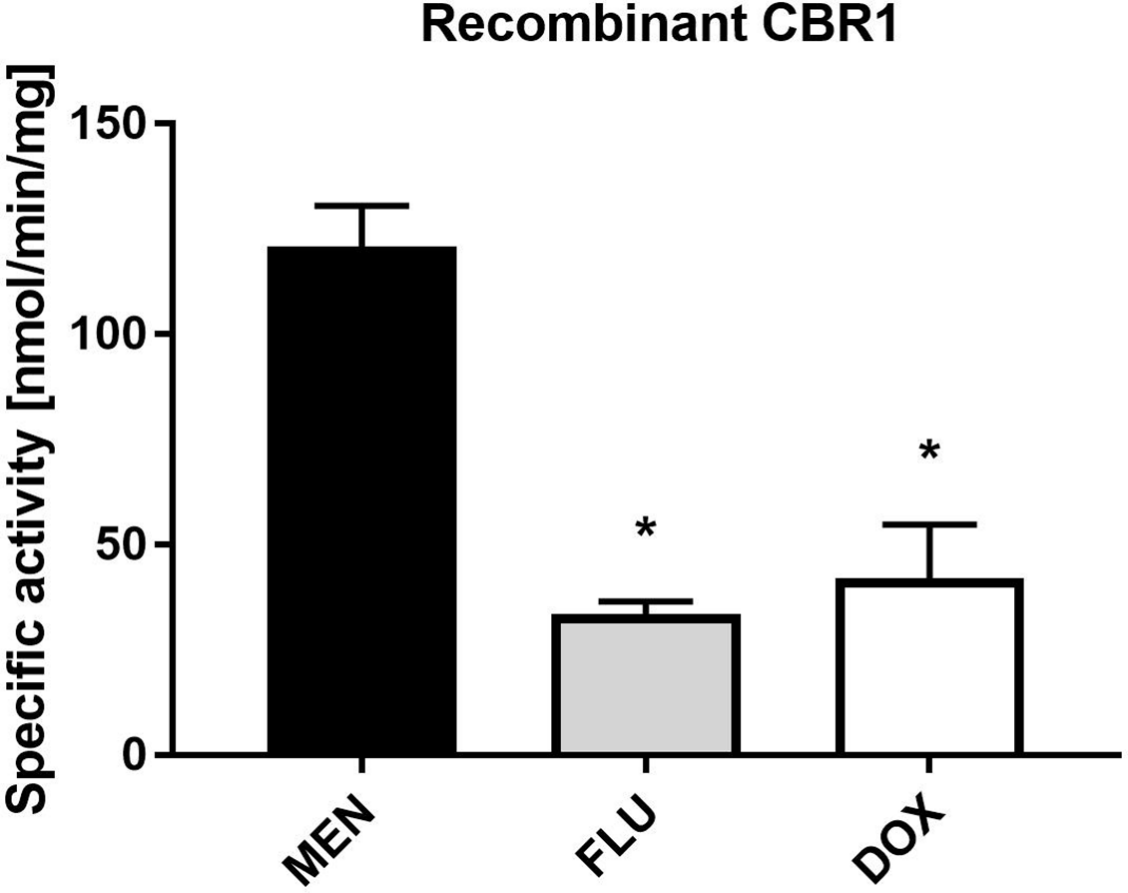
Specific activity of human recombinant CBR1 toward menadione (MEN), flubendazole (FLU), and doxorubicin (DOX). CBR1 was incubated with 10 µM substrates (MEN, FLU, and DOX). The values represent the mean ± standard deviation of three independent experiments. *Significantly different compared with MEN.

The specific activity of human recombinant CBR1 toward MEN, FLU and DOX as substrates was measured using the spectrophotometric detection of NADPH consumption. The specific activity of CBR1 was the highest toward MEN, and comparable to FLU and DOX (**Figure 5**).

### Inter-Individual Variability of CBR1 Level in Human Hepatic Cytosols

**Figure 6:**
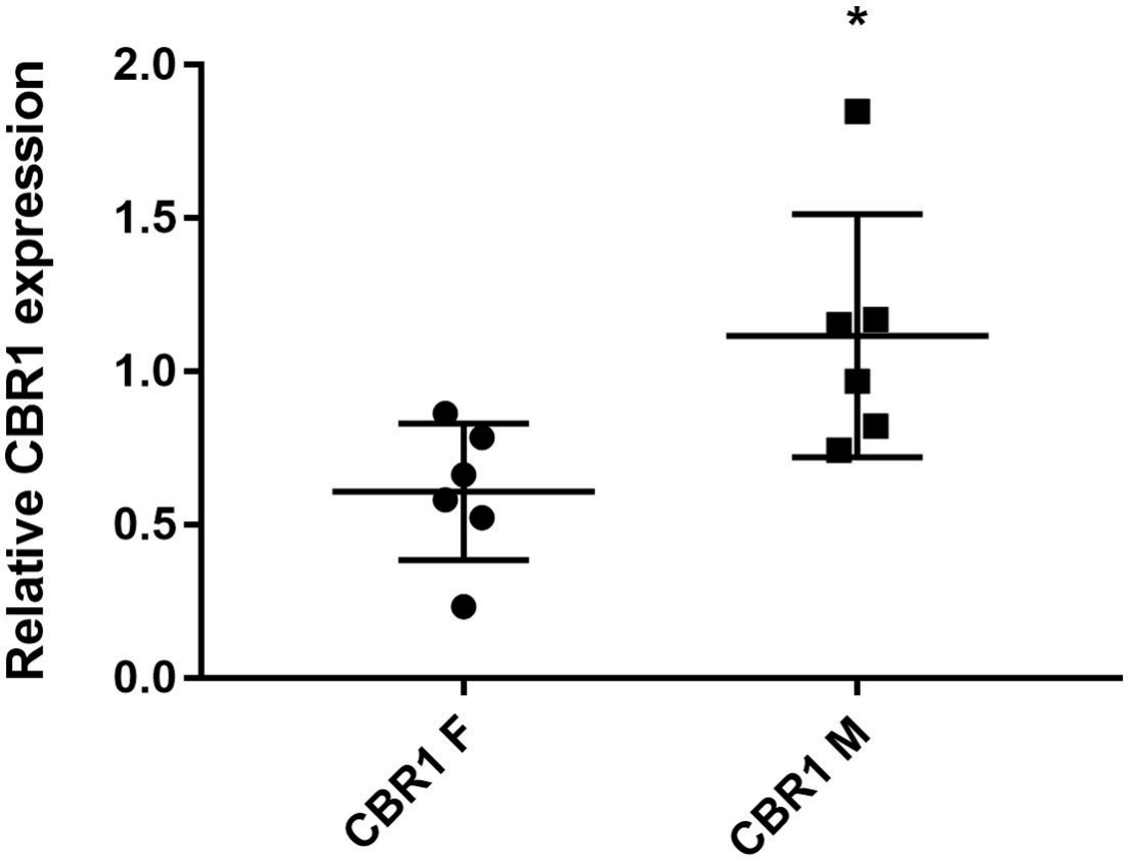
The levels of CBR1 in liver cytosol from individual human samples [F, female (n = 6); M, male (n = 6)]. Protein levels were measured in two independent experiments. *Significantly different compared with F.

The level of CBR1 was analyzed using immunoblotting. The results of densitometric quantification are presented in **Figure 6**. The CBR1 levels were significantly higher in the male patients than in female patients. Relatively low inter-individual variability was observed.

### Flubendazole Effect on Doxorubicin Reduction in Cytosol From Human Liver and Cancer Cells SW480

The potential of FLU to inhibit reduction of the anticancer drug DOX (known CBR1 substrate) was tested in the human liver and in cancer cells SW480. Cytosolic fractions were incubated with DOX (20 µM), NADPH, and FLU (0, 2, or 10 µM). The amount of formed doxorubicinol was measured. The results summarized in **Table 4** show that FLU in the tested concentrations inhibit DOX reduction neither in the human liver nor in cancer cells SW480.

**Table 4:** Flubendazole effect on doxorubicin (20 µM) reduction in the pooled human liver cytosol of both sexes and in cancer cells SW480.

Flubendazole	Doxorubicinol—cytosol	Doxorubicinol—SW480
0 µM	100.0 ± 5.8%	100.0 ± 4.6%
2 µM	98.0 ± 3.4%	97.6 ± 3.4%
10 µM	91.4 ± 4.9%	96.7 ± 2.7%

The values are expressed as the percent of the residual activity (the mean ± standard deviation of three independent experiments).

## Discussion

In human medicine, FLU is used only for treatment of gut-residing nematodes. However, the anti-proliferative, anti-metastatic, and anticancer efficacy of FLU have been intensively studied, and many promising results have recently been reported (Canova et al., 2018; Kralova et al., 2018; Oh et al., 2018). Moreover, the macrofilaricidal (Geary et al., 2019) and the anti-fungal activity of FLU have been revealed, and the efficacy of FLU in the treatment of cryptococcal meningoencephalitis has been shown (Nixon et al., 2018). For these reasons, the repurposing of FLU for other indications and early increase of its use in human therapy are very likely. From this point of view, the study of the fate of FLU in humans is of great importance. Nevertheless, knowledge about FLU metabolism in human has been until now limited. In 2017, Mate et al. (2017) reported that FLU was reduced and hydrolyzed in pooled human liver microsomes, leading to the formation of FLUR and FLUH. Surprisingly, FLUR was the only metabolite of FLU detected in human liver slices from four patients using ultrasensitive UHPLC/MS (Stuchlikova et al., 2018). In the present study, 12 individual and pooled human liver microsomes as well as cytosols were used, but no hydrolyzed FLU was found. Based on this finding, we suppose that FLUR is the only FLU metabolite formed in the human liver.

With the aim of finding out more about FLU reduction, FLUR formation in human liver microsomes and cytosols was compared and the preference of coenzymes was tested. As FLU is a pro-chiral substrate and its carbonyl reduction can lead to the formation of two stereoisomers of FLUR, chiral analysis was used. However, only one FLUR enantiomer, identified as (+)-FLUR, was detected in all the samples, indicating strict stereospecificity of FLU reduction in the human liver. Previously, the marked but not strict stereospecificity of FLU reduction was reported in the sheep, where the formation of (+)-FLUR also dominated (Nobilis et al., 2008; Krizova et al., 2009). Concerning subcellular localization and coenzyme preference of FLU reduction, cytosol is much more important than microsomes, and NADPH is a more effective coenzyme than NADH in the human liver.

Consequently, the kinetics of FLU reduction were studied in pooled human liver cytosols. Although poor FLU water solubility allowed only the use of a 10-µM concentration as the highest in incubation mixtures, the apparent kinetic parameters and intrinsic clearance of FLU reduction could be calculated. The obtained data indicate that the extent of FLU reduction in the human liver is relatively low. The slow and less effective reduction of FLU was also observed in precise-cut human liver slices (Stuchlikova et al., 2018). Based on these findings, relatively slow FLU deactivation and a low first-pass effect can be expected in humans, but this should be verified in pharmacokinetic studies.

In further experiments, FLU reduction was studied in individual human liver cytosols (from six male and six female patients) to evaluate possible sex differences and inter-individual differences. Although inter-individual differences were relatively low, a significant sex difference was revealed: reductases from the male cytosols reduced FLU more effectively than the female ones.

When we considered which enzyme reduced FLU in human cytosol, carbonyl reductase 1 (CBR1) was the main candidate. CBR1, the cytosolic enzyme preferring the NADPH coenzyme, plays an essential role in deactivation of ketones and aldehydes. CBR1 has a broad substrate specificity, and this enzyme is involved in the metabolism of many clinically important drugs (Shi and Di, 2017). First, the participation of CBR1 in FLU reduction was tested using the specific inhibitors MEN and LUT. MEN is very good substrate of CBR1 and it is often used as competitive inhibitor (Gonzalez-Covarrubias et al., 2008). Many flavonoids, including LUT, have been shown to inhibit CBR1 (Arai et al., 2015; Bousova et al., 2015). In our experiment, both inhibitors significantly decreased FLUR formation in human liver cytosols. Additionally, the participation of CBR1 in FLU reduction was proved using human recombinant enzyme. However, the activity of recombinant CBR1 to FLU was significantly lower than to MEN, indicating that FLU is reduced by CBR1 but it is not very good substrate for this enzyme. When we compared the CBR1 level in liver cytosols from six male patients and six female patients, significantly higher levels of CBR1 were found in male patients. This finding is in a good agreement with higher FLU cytosolic reduction in male patients than female patients. This result highlighted the central role of CBR1 in FLU reduction; however, partial participation of other carbonyl reducing enzymes cannot be excluded.

The present study was also aimed at evaluating possible FLU interactions with other drugs bearing a carbonyl group. Our results showed that human recombinant CBR1 had similar activity toward FLU and DOX, a wildly used cytostatic drug and known CBR1 substrate (Gonzalez-Covarrubias et al., 2008). If FLU and DOX are both substrates of the same reductase(s), there was a certain probability that FLU could act as a competitive inhibitor of DOX reduction. If FLU is able to inhibit DOX reduction, it may decrease DOX deactivation in the human liver and cancer cells with possible pharmacological and toxicological consequences. Based on this fact, we decided to test the effect of FLU on DOX reduction in pooled human liver cytosols as well as in cytosols from colon adenocarcinoma cells SW480. These cells were selected due to the results of a previous study which showed the high ability of SW480 cells to reduce FLU in comparison to other cancer cell lines (Stuchlikova et al., 2018). Our results showed that FLU did not inhibit reduction of DOX either in the human liver or cancer cells.

In conclusion, FLU is predominantly reduced in cytosol and NADPH is the preferred coenzyme. Carbonyl reduction of FLU is strictly stereospecific and CBR1 is the main FLU reductase in human liver. Comparing individuals, a higher level of CBR1 and more effective FLU reduction was observed in male patients than in female patients, but overall inter-individual variability was low. As FLU did not inhibit carbonyl reduction of DOX in the liver nor in cancer cells, interactions of these drugs is not expected. With respect to the relatively low affinity and activity of human liver reductases toward FLU, the risk of FLU interactions with other carbonyl bearing drugs is unlikely. All these findings support FLU repurposing for other indications and its wide use.

## Data Availability Statement

The data sets generated during and/or analyzed during the current study are available from the corresponding author on request.

## Ethics Statement

This study was carried out in accordance with the recommendations of the Declaration of Helsinki and Good Clinical Practice Guidelines. The protocol was approved by the Ethics Committee of University Hospital Hradec Králové, Czechia (project “Collection of liver tissue for isolation of hepatocytes, preparation of precision liver slices and subcellular fractions,” No. 201703 S14P). Small pieces of healthy liver tissue were obtained from patients undergoing liver surgery due to a tumor. All patients gave written informed consent in accordance with the Declaration of Helsinki.

## Author Contributions

LS and BS designed the research study. VKu, JH, JŠ, ZŠ, and AS performed the study. VKr performed the incubation of SW480 cells. VKu and BS analyzed the data. LS and BS wrote the manuscript.

## Funding

This work was supported by the project EFSA-CDN (No. CZ.02.1.01/0.0/0.0/16_019/0000841) co-funded by ERDF and by the Charles University in Prague (Progres Q40/1, Progres Q42, SVV 260 416).

## Conflict of Interest Statement

The authors declare that the research was conducted in the absence of any commercial or financial relationships that could be construed as a potential conflict of interest.
